# Supplementary material for: Prediction models used in the progression of chronic kidney disease: A scoping review
Source: PLoS One. 2022 Jul 26;17(7):e0271619. doi: 10.1371/journal.pone.0271619 (PMC9321365; doi:10.1371/journal.pone.0271619)

**S1 Appendix. Detailed search strategy per database.**

**Medline concept grid**

| **Medline advanced search** | | | | | | | |
| --- | --- | --- | --- | --- | --- | --- | --- |
| **CONCEPT GRID** | **Kidney disease** | **A**  **N**  **D** | **Disease progression** | **A**  **N**  **D** | **Techniques** | **A**  **N**  **D** | **Outcomes** |
| **Keywords** | chronic kidney disease* **OR**  chronic renal disease* **OR**  CKD **OR**  kidney disease* **OR**  kidney failure |  | (progress* adj7 (CKD or disease)).ti,ab. |  | deep learning **OR**  machine learning **OR**  artificial intelligence **OR**  algorithms **OR**  prediction model* **OR**  statistic* model* |  | End stage renal disease **OR**  ESRD **OR**  Transplant* **OR**  Hemodialysis **OR**  Hospitali?ation **OR**  Mortality **OR**  Morbidity **OR**  Heart failure **OR**  Stroke |
| **OR** | |  | **OR** |  | **OR** |  | **OR** |
| **Subject headings**  **(MeSH Terms)**  **Medline** | Renal Insufficiency, Chronic/ **OR**  Kidney Failure, Chronic/ **OR**  Diabetic Nephropathies/ |  | Disease Progression/ |  | Artificial Intelligence/ **OR**  Big data/ **OR**  machine learning/ **OR**  algorithms/ **OR**  Models, Statistical/ |  | Dialysis/ **OR**  Peritoneal Dialysis/ **OR**  Renal Dialysis/ **OR**  Kidney Transplantation/ **OR**  Cardiovascular Diseases/ **OR**  Hypertension/ **OR**  Coronary Artery Disease/ **OR**  Coronary Disease/ **OR**  Hospitalization/ **OR**  Heart failure/ **OR**  Stroke/ |

**MEDLINE** **search**
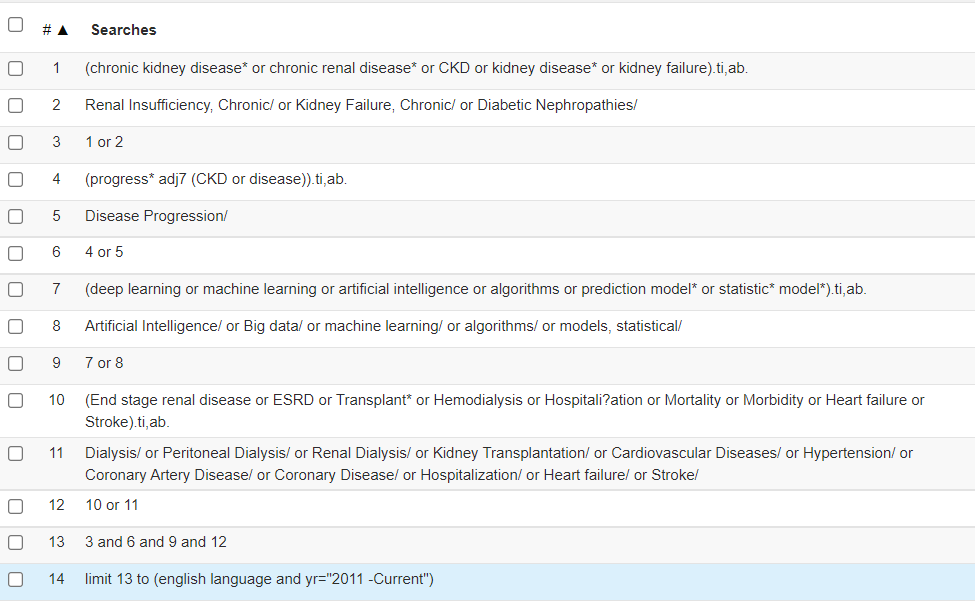


**EMBASE concept grid**

| **EMBASE advanced search** | | | | | | | |
| --- | --- | --- | --- | --- | --- | --- | --- |
| **CONCEPT GRID** | **Kidney disease** | **A**  **N**  **D** | **Disease progression** | **A**  **N**  **D** | **Techniques** | **A**  **N**  **D** | **Outcomes** |
| **Keywords** | chronic kidney disease* **OR**  chronic renal disease* **OR**  CKD **OR**  kidney disease* **OR**  kidney failure |  | (progress* adj7 (CKD or disease)).ti,ab. |  | deep learning **OR**  machine learning **OR**  artificial intelligence **OR**  algorithms **OR**  prediction model* **OR**  statistic* model* |  | End stage renal disease **OR**  ESRD **OR**  Transplant* **OR**  Hemodialysis **OR**  Hospitali?ation **OR**  Mortality **OR**  Morbidity **OR**  Heart failure **OR**  Stroke |
| **OR** | |  | **OR** |  | **OR** |  | **OR** |
| **Subject headings**  **(MeSH Terms)**  **Medline** | Chronic kidney failure/ **OR**  Diabetic Nephropathy/ |  | Disease exacerbation/ |  | Artificial Intelligence/ **OR**  Big data/ **OR**  machine learning/ **OR**  algorithm/ **OR**  statistical model/ |  | Dialysis/ **OR**  Peritoneal Dialysis/ **OR**  hemodialysis/ **OR**  Kidney Transplantation/ **OR**  Cardiovascular disease/ **OR**  Hypertension/ **OR**  Coronary Artery Disease/ **OR**  Hospitalization/ **OR**  Heart failure/ **OR**  Cerebrovascular accident/ |

**EMBASE search**


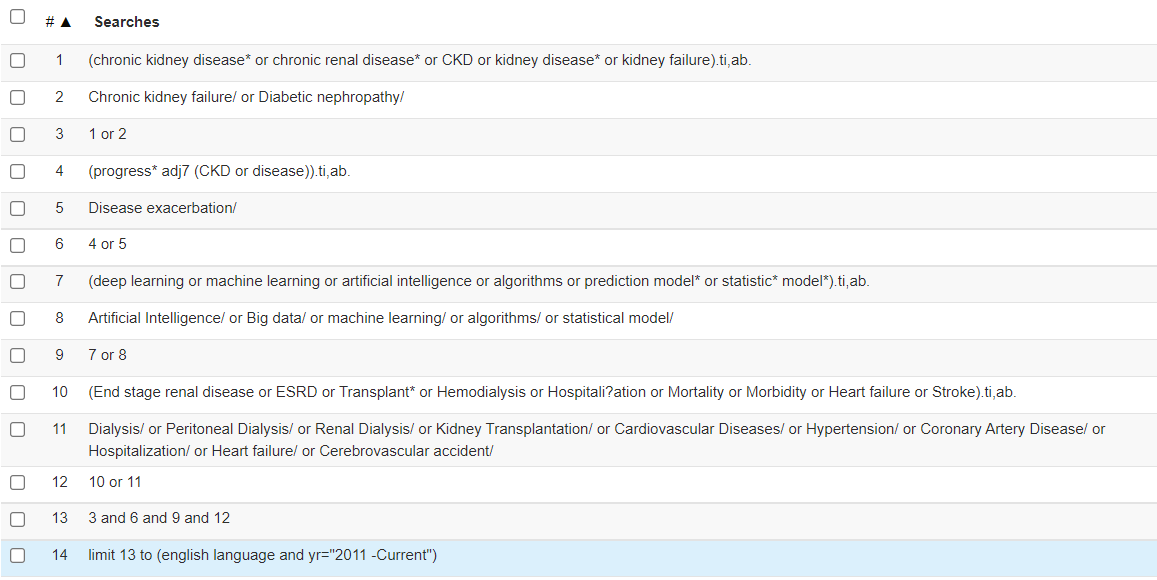


**CINAHL concept grid**

| **CINAHL advanced search** | | | | | | | |
| --- | --- | --- | --- | --- | --- | --- | --- |
| **CONCEPT GRID** | **Kidney disease** | **A**  **N**  **D** | **Disease progression** | **A**  **N**  **D** | **Techniques** | **A**  **N**  **D** | **Outcomes** |
| **Keywords** | chronic kidney disease* **OR**  chronic renal disease* **OR**  CKD **OR**  kidney disease* **OR**  kidney failure |  | (progress* adj7 (CKD or disease)).ti,ab. |  | deep learning **OR**  machine learning **OR**  artificial intelligence **OR**  algorithms **OR**  prediction model* **OR**  statistic* model* |  | End stage renal disease **OR**  ESRD **OR**  Transplant* **OR**  Hemodialysis **OR**  Hospitali?ation **OR**  Mortality **OR**  Morbidity **OR**  Heart failure **OR**  Stroke |
| **OR** | |  | **OR** |  | **OR** |  | **OR** |
| **Subject headings**  **(MeSH Terms)**  **Medline** | Renal Insufficiency, Chronic/ **OR**  Kidney Failure, Chronic/ **OR**  Diabetic Nephropathies/ |  | Disease Progression/ OR  Disease exacerbation/ |  | Artificial Intelligence/ **OR**  Data Analytics/ **OR**  Machine learning/ **OR**  Algorithms/ **OR**  Models, Statistical/ |  | Dialysis/ **OR**  Peritoneal Dialysis/ **OR**  Hemodialysis/ **OR**  Kidney Transplantation/ **OR**  Cardiovascular Diseases/ **OR**  Hypertension/ **OR**  Coronary Disease/ **OR**  Hospitalization/ **OR**  Heart failure/ **OR**  Stroke/ |

**CINAHL search**


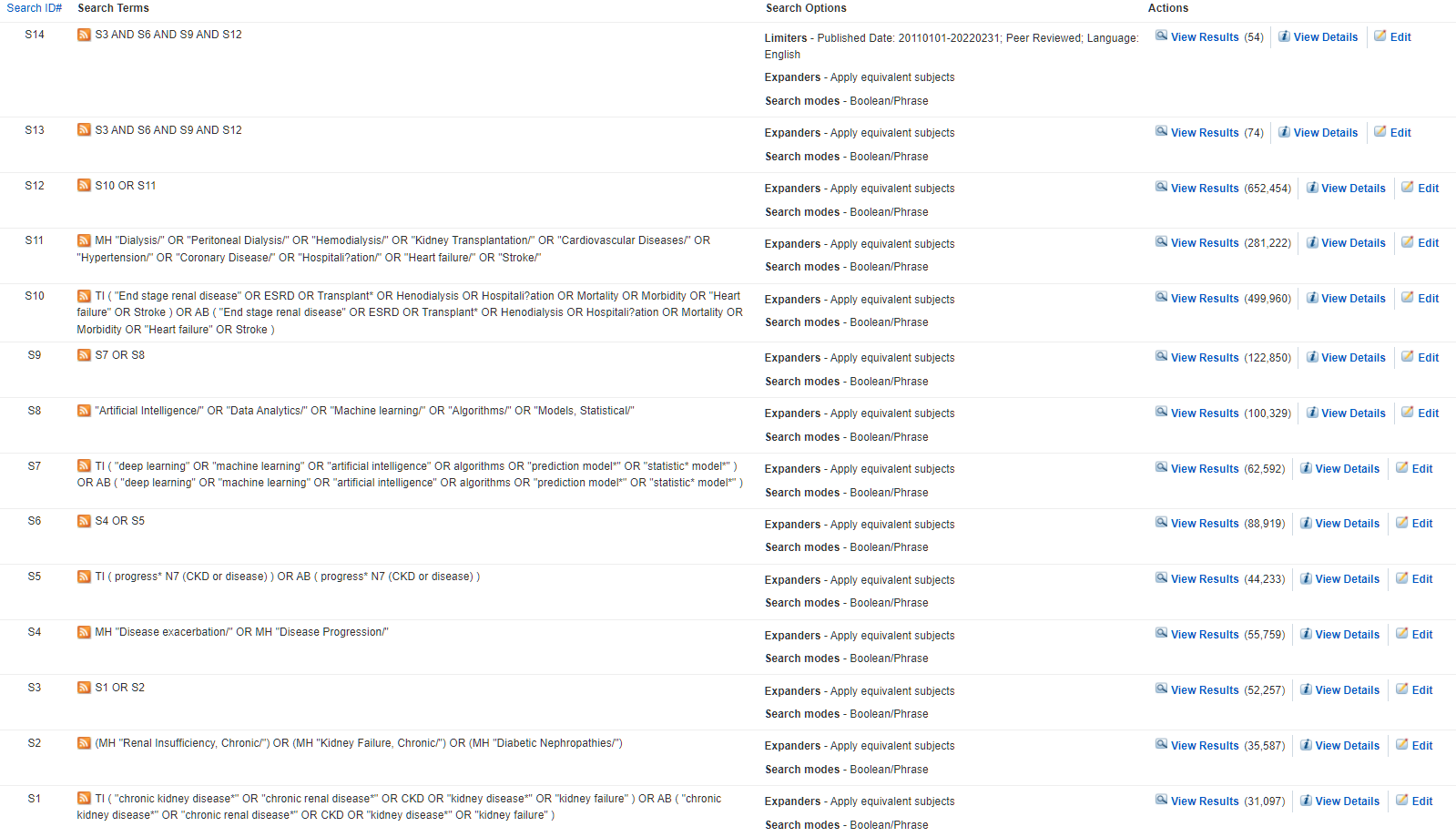


**Scopus search**


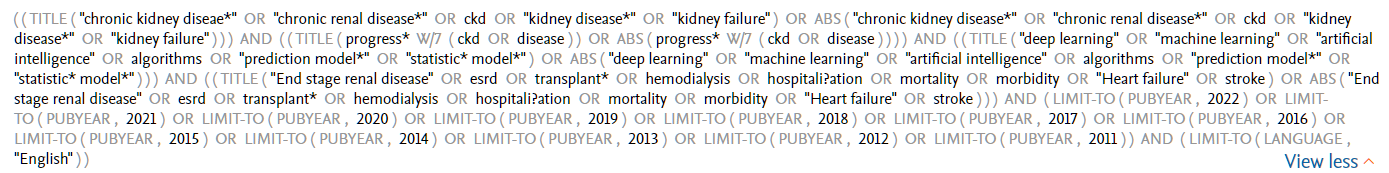

Supplement: S1 Appendix — Concept grids and search histories for Medline, EMBASE, CINAHL and Scopus. (DOCX) [file pone.0271619.s002.docx]
